# A Case of Advanced Basal Cell Carcinoma of the Head and Neck: Diagnostic Challenges and Sequential Systemic Therapy

**DOI:** 10.7759/cureus.118413

**Published:** 2026-10-22

**Authors:** Ivan Bivolarski, Tanio Stefanov, Martin Dimitrov, Anita R Hvarchilkova-Baycheva, Yavor S Pavlov, Stanislav Marangozov

**Affiliations:** 1 Medical Oncology, Integrated Oncology Centre Burgas, Burgas, BGR; 2 Surgical Oncology, Hospital Burgasmed, Burgas, BGR; 3 Visceral Surgery, Kreiskrankenhaus Greiz, Greiz, DEU; 4 Medical Oncology, Complex Oncology Center Veliko Tarnovo, Veliko Tarnovo, BGR; 5 Pathology, Multiprofile Hospital for Active Treatment "Burgasmed", Burgas, BGR; 6 General and Thorax Surgery, UMBAL (Universitetska Mnogoprofilna Bolnitsa za Aktivno Lechenie) Burgasmed, Burgas, BGR

**Keywords:** 5 fluorouracil, basal cell carcinoma, basaloid carcinoma, basaloid variant of squamous cell carcinoma, cemiplimab, cisplatin, sequential systemic therapy, vismodegib

## Abstract

Advanced cutaneous malignancies with basaloid morphology can present diagnostic and therapeutic challenges, particularly when pathological interpretations differ and disease progresses during systemic treatment. We describe the clinical course of a 53-year-old woman with an extensive, unresectable cutaneous tumor involving the posterior neck and occipital region, accompanied by bilateral cervical lymphadenopathy and additional clinically suspected cutaneous deposits. The initial diagnosis was trichoblastic carcinoma, whereas expert pathological review favored basal cell carcinoma. The extent of disease precluded surgery and definitive radiotherapy. First-line vismodegib was initiated, and palliative radiotherapy delivering 30 Gy in 10 fractions to the occipital lesions and bilateral cervical lymph nodes was completed one year later. Clinical progression was documented three months after completion of radiotherapy; one month after this, MRI demonstrated extensive infiltration involving the left parotid gland and adjacent pharyngeal spaces. Second-line cisplatin and 5-fluorouracil were subsequently administered. Follow-up MRI six months after the earlier MRI demonstrated substantial tumor volume reduction. PET/CT three months later showed a heterogeneous response compared with a year earlier, including regression at several previously involved sites and newly apparent metabolically active right axillary lymph nodes. A subsequent biopsy was reported as basaloid carcinoma. The patient later experienced clinical deterioration, received one cycle of cemiplimab, and died before further treatment or response assessment. This case illustrates the importance of pathological clarification, longitudinal imaging, and careful interpretation of treatment response during sequential systemic therapy for advanced basal cell carcinoma.

## Introduction

Basal cell carcinoma (BCC) is the most common skin malignancy and is usually managed successfully with local treatment. Although metastasis is rare, advanced BCC can cause extensive tissue destruction, functional impairment, and substantial morbidity. Tumors involving the head and neck are particularly challenging when their extent or anatomical location precludes curative surgery or radiotherapy. Accurate histopathological diagnosis and multidisciplinary assessment are essential for determining disease extent and selecting an appropriate treatment strategy [1].

Systemic treatment options have expanded with the introduction of Hedgehog pathway inhibitors. Vismodegib, a Smoothened inhibitor, demonstrated clinically meaningful activity in both locally advanced and metastatic BCC in the pivotal ERIVANCE study [2]. However, disease progression or intolerance may necessitate subsequent therapy. Current European recommendations position Hedgehog pathway inhibitors as principal systemic treatments for advanced BCC and recommend anti-programmed cell death protein 1 (PD-1) therapy following progression, intolerance, or contraindication to these agents [1]. Cemiplimab has demonstrated antitumor activity in locally advanced BCC previously treated with a Hedgehog pathway inhibitor [3], with subsequent phase II results supporting its activity in metastatic BCC in the same treatment setting [4]. Despite these advances, management remains complex in patients with extensive symptomatic disease, declining performance status, or uncertain pathological classification, requiring integration of pathological findings, longitudinal imaging, clinical condition, and goals of care.

We report the case of a woman with advanced BCC of the head and neck whose clinical course included differing pathological descriptions, progression during vismodegib treatment, substantial MRI-documented regression following cisplatin and 5-fluorouracil, a subsequent heterogeneous PET/CT response, and histologically confirmed lymph-node involvement by basaloid carcinoma. She later received a single cycle of cemiplimab before her death, precluding further treatment and response assessment. This case highlights the diagnostic and therapeutic challenges encountered during sequential systemic treatment and the importance of distinguishing site-specific regression from overall disease control.

## Case presentation

A 53-year-old woman with a documented history of cardiac arrhythmia presented in November 2024 with progressively worsening neck pain and swelling. Clinical examination and ultrasonography identified an extensive cutaneous lesion involving the posterior neck, multiple enlarged bilateral cervical lymph nodes measuring more than 3 cm, and additional cutaneous lesions, including lesions on the upper back. Several of the cutaneous lesions were reported to exceed 4 cm. A biopsy was performed in the same month, and the initial pathological assessments included trichoblastic carcinoma and solid-type BCC with focal trichilemmal differentiation. Expert pathological review at a referral institution was reported to favor BCC.

Multidisciplinary assessment determined that the disease was unresectable and unsuitable for definitive radiotherapy because of its extensive distribution. First-line treatment with oral vismodegib at a dose of 150 mg once daily was initiated in January 2025.

An 18F-fluorodeoxyglucose (18F-FDG) PET/CT examination performed in February 2025 demonstrated a large, metabolically active soft-tissue tumor involving the left facial and cervical regions, with extension above the skin surface and a maximum standardized uptake value (SUVmax) of 12.08 (Figure 1).

**Figure 1:**
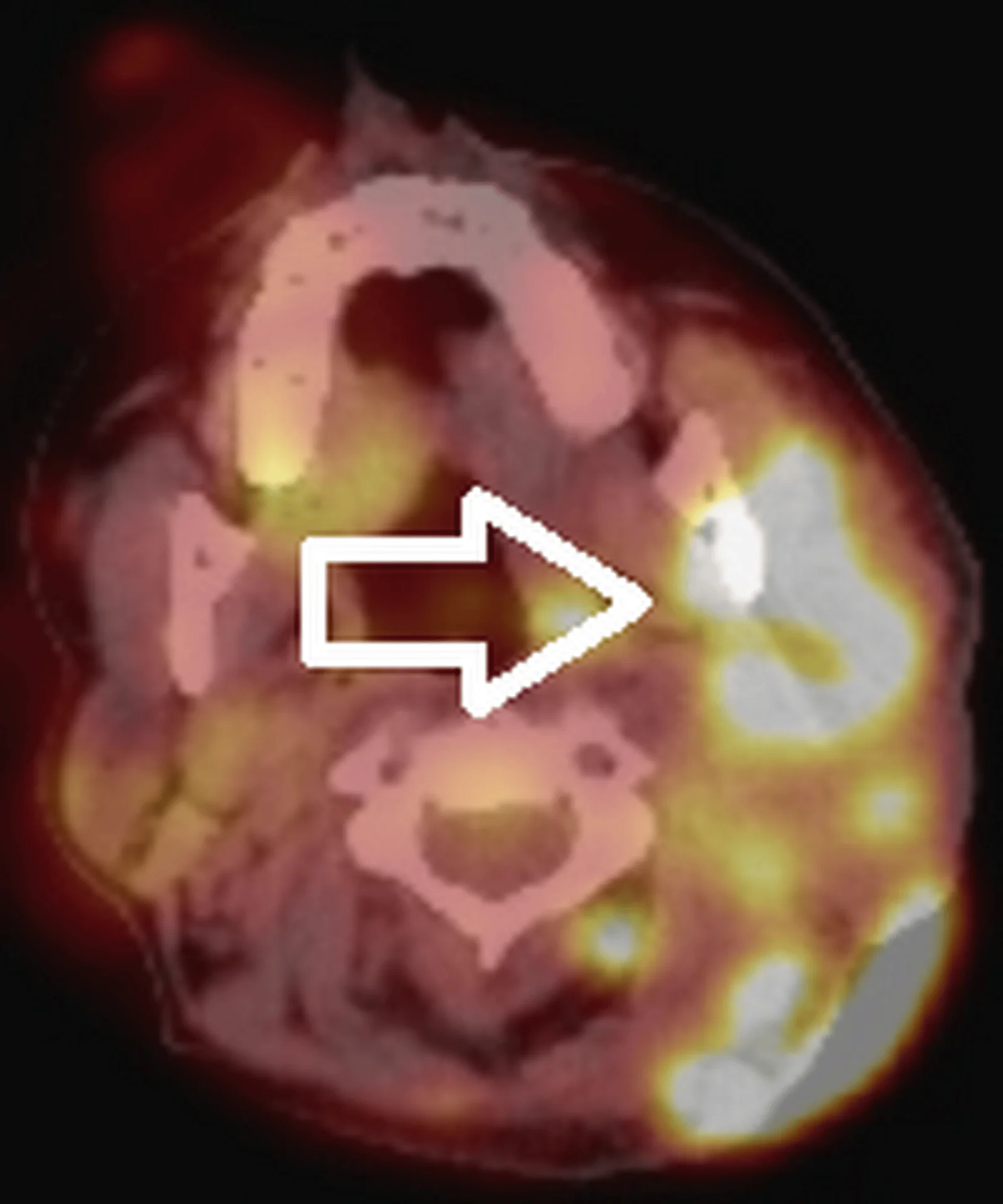
Initial 18F-FDG PET/CT performed in February 2025 (axial view) demonstrating intense FDG uptake in the left cervical soft-tissue tumor and adjacent cervical lymph nodes. 18F-FDG: 18F-fluorodeoxyglucose

The same examination showed enlarged, metabolically active bilateral cervical lymph nodes (Figure 2), including nodes along the spinal accessory chains. The nodes formed conglomerates on the left and had SUVmax values of up to 22.72. Metabolically active left supraclavicular and axillary lymph nodes had SUVmax values of up to 20.14. A poorly defined, metabolically active soft-tissue finding in the left tonsillar region had an SUVmax of 15.97; histological clarification was recommended. An additional focus in the anterior aspect of the left second rib had an SUVmax of 5.53 without a definite corresponding morphological abnormality, and imaging follow-up was advised. No metabolically active pulmonary or hepatic lesions or metabolically active abdominal or pelvic lymph nodes were reported. The clinically described cutaneous lesions on the upper back did not show increased metabolic activity on this examination.

**Figure 2:**
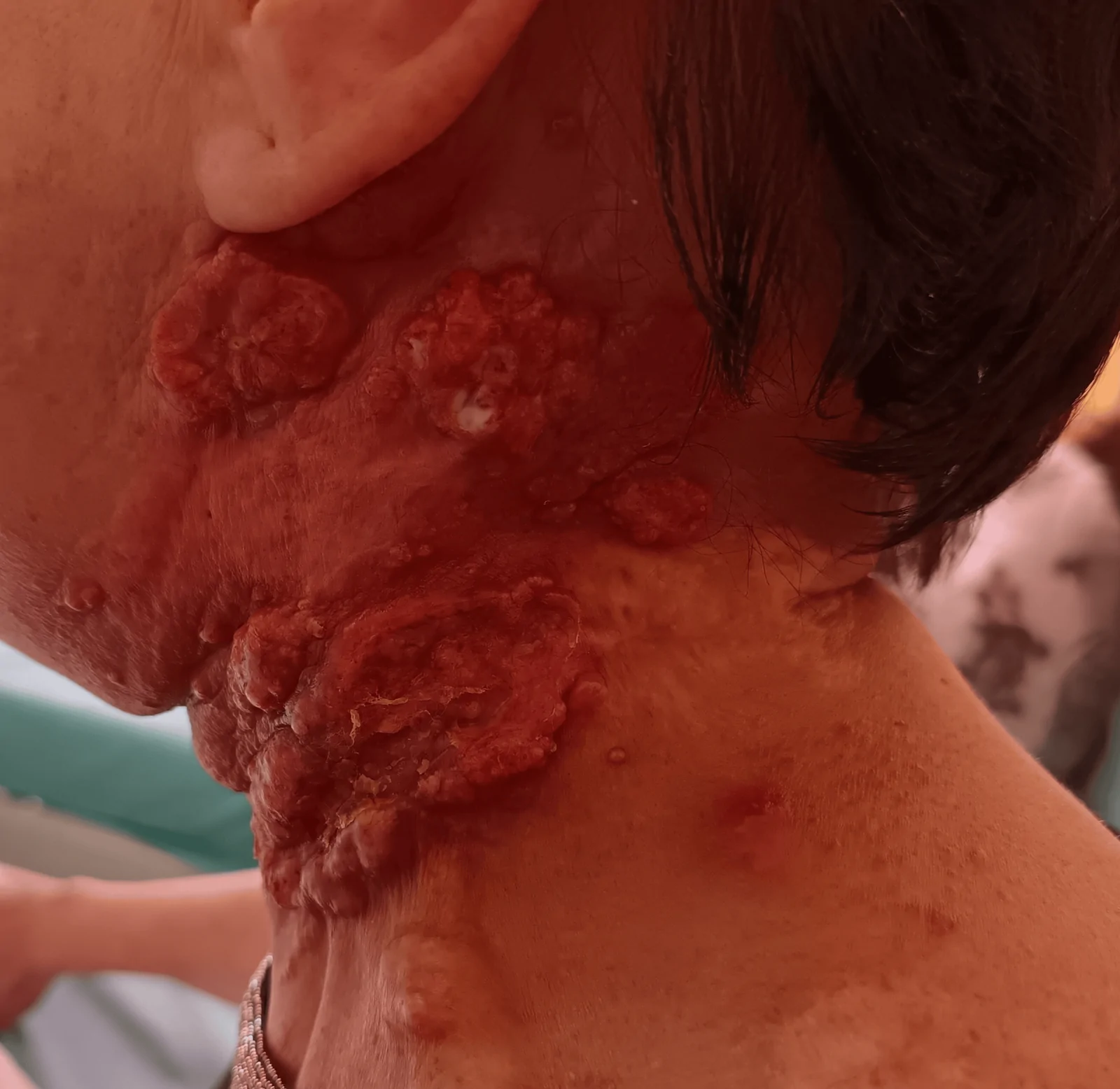
Clinical appearance of the left cervical and infra-auricular regions showing multiple raised erythematous nodules and plaques, some confluent, with prominent ulceration and crusting.

Because of marked pain and discomfort associated with the occipital lesion, palliative radiotherapy was delivered to the occipital cutaneous lesions and bilateral cervical lymph nodes in February 2025. A total dose of 30 Gy was administered in 10 fractions of 3 Gy using 6-MV photons. Vismodegib treatment was continued.

In May 2025, clinical progression was documented, with newly apparent cervical lesions. Contrast-enhanced magnetic resonance imaging (MRI) of the neck performed in June 2025 demonstrated an extensive lobulated cutaneous and subcutaneous tumor along the left side of the neck. The tumor extended inferiorly to approximately the middle third of the neck and superiorly toward the submandibular region and left parotid gland. Infiltration of the deep parotid components and extension toward the nasopharyngeal and retropharyngeal structures were described. Superior extension involved the occipital cutaneous and subcutaneous tissues. The tumor demonstrated prominent contrast enhancement and heterogeneous areas of restricted diffusion. Bilateral enlarged cervical lymph nodes were also present, with the largest reported right-sided lymph node measuring 19 × 12 mm.

Following multidisciplinary review, second-line chemotherapy with cisplatin and 5-fluorouracil was selected, with the first cycle planned for June 2025. Seven cycles were subsequently administered.

Follow-up neck MRI was performed in December 2025 and, compared with the June examination, demonstrated a substantial reduction in the volume of the occipital cutaneous and subcutaneous tumor, the superficial and deep parotid involvement, and the retropharyngeal and left lateral nasopharyngeal abnormalities. Areas of restricted diffusion had also decreased in size and conspicuity. Residual disease persisted, including a right parasagittal nasopharyngeal component. The radiologist concluded that there had been a significant reduction in tumor volume.

Repeat 18F-FDG PET/CT performed in March 2026 and compared with the February 2025 examination demonstrated a heterogeneous treatment response. The left tonsillar finding showed a decrease in SUVmax from 15.97 to 8.11. The left facial and cervical soft-tissue tumor was substantially smaller, although its SUVmax increased from 12.08 to 13.45. Bilateral cervical lymph nodes, including those along the spinal accessory chains, decreased in size, with SUVmax declining from 22.72 to 10.63. The previously described left axillary lymph nodes persisted, although their SUVmax decreased from 20.14 to 8.08. Newly apparent metabolically active right axillary lymph nodes were also reported. No metabolically active pulmonary, hepatic, skeletal, abdominal nodal, or pelvic nodal lesions were identified. Diffuse bone marrow activation was noted. Because the comparison encompassed treatment with vismodegib, radiotherapy, and chemotherapy, the observed PET/CT changes could not be attributed exclusively to a single treatment modality.

To obtain histological confirmation of the nodal disease, the patient was admitted for surgical biopsy in March 2026. A pathologically enlarged left axillary lymph node was excised under local anesthesia. The perioperative course was uncomplicated. Histopathological examination demonstrated a 1.3-cm lymph node containing metastatic basaloid carcinoma with infiltration of the nodal capsule. A separate 1.6-cm tissue fragment was also infiltrated by basaloid carcinoma. These findings provided histological confirmation of lymph node involvement by basaloid carcinoma. However, because a complete microscopic description and immunohistochemical profile were unavailable, the findings did not independently establish histological transformation (Figure 3).

**Figure 3:**
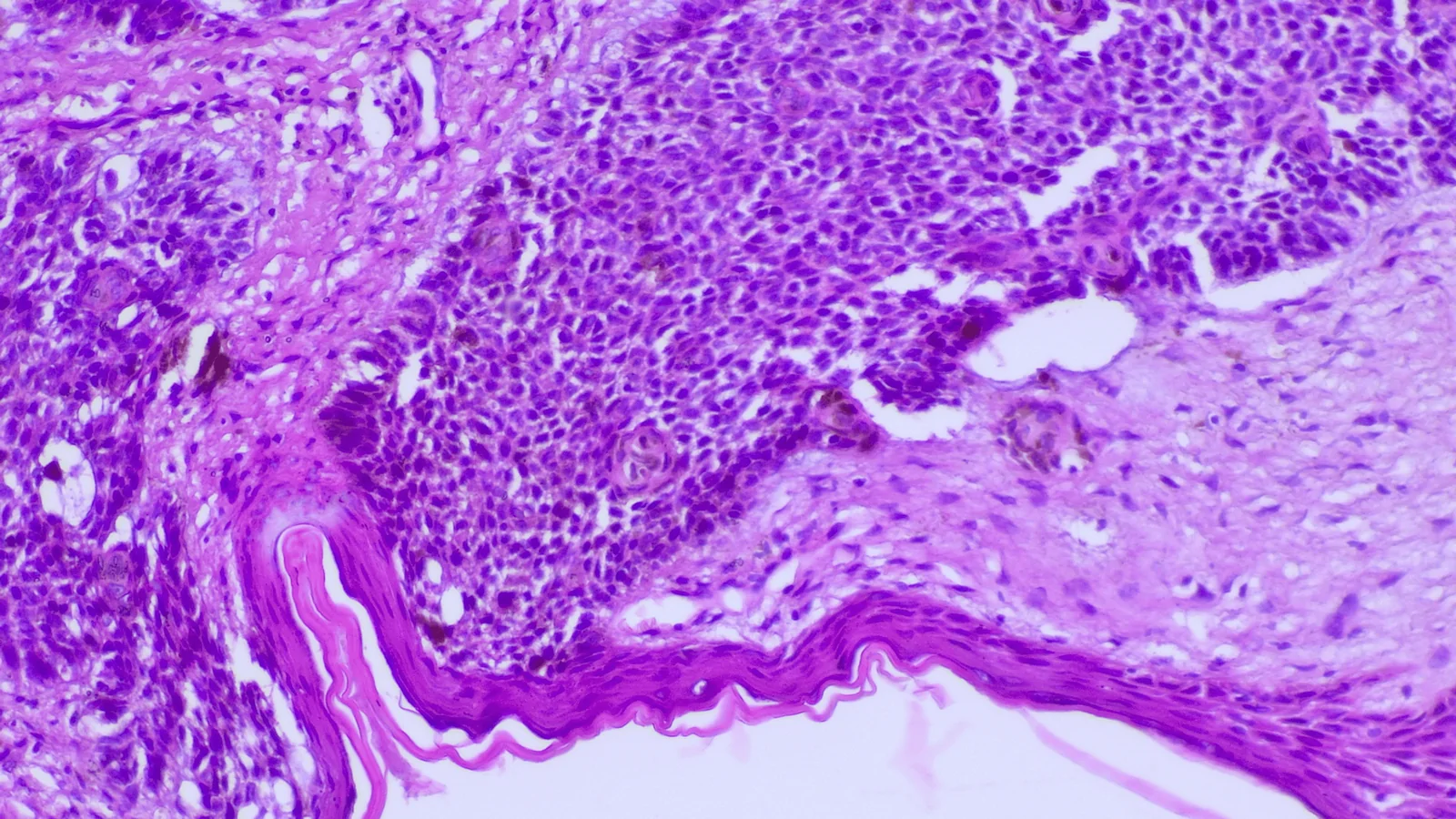
Repeat biopsy showing nests of basaloid tumor cells within fibrous stroma, with an adjacent inflammatory infiltrate (hematoxylin and eosin staining).

By May 2026, the patient had experienced further clinical deterioration and was admitted for symptomatic and palliative care. Her Eastern Cooperative Oncology Group (ECOG) performance status was 3. She presented with marked fatigue, nausea, vomiting, anorexia, and severe pain. Physical examination revealed a firm, deeply fixed left cervical lymph-node conglomerate and multiple confluent cutaneous and subcutaneous lesions involving the left lateral and posterior cervical regions and extending onto the left upper back. The cutaneous lesions were ulcerated, and a firm left preauricular swelling was also present.

Laboratory investigations performed in May 2026 showed a hemoglobin concentration of 94 g/L (laboratory reference interval: 135-180 g/L), a mean corpuscular volume of 109.8 fL (82-96 fL), a platelet count of 545 × 10⁹/L (100-440 × 10⁹/L), and a serum albumin level of 28 g/L (34-54 g/L). These findings indicated macrocytic anemia, thrombocytosis, and hypoalbuminemia. Anemia may have contributed to fatigue and reduced functional capacity, while thrombocytosis could have reflected a reactive response to malignancy or inflammation. Hypoalbuminemia was consistent with systemic illness and nutritional compromise, although its cause was not established. The hemoglobin concentration decreased to 87 g/L a week later and subsequently increased to 111 g/L four days after this; the reason for this increase is not known. Supportive management included analgesics, antiemetics, intravenous fluids, parenteral nutritional support, and topical wound care.

The patient subsequently received one cycle of cemiplimab. No further treatment could be administered because the patient died thereafter in July 2026, 106 days after the biopsy procedure. No post-treatment response assessment was performed. Consequently, no conclusion could be drawn regarding the efficacy of cemiplimab in this patient or any causal relationship between its administration and her death.

## Discussion

This case illustrates the complexity of managing advanced BCC of the head and neck when extensive local infiltration, differing pathological descriptions, and heterogeneous responses to sequential treatment coexist. Although substantial radiographic regression was documented during the treatment course, residual metabolically active disease, histologically confirmed nodal involvement, and subsequent clinical deterioration demonstrated that tumor reduction at individual sites did not translate into sustained overall clinical benefit.

An important feature of this case was the variation in pathological terminology across the available records. The initial tumor was variously described as trichoblastic carcinoma and solid-type BCC with focal trichilemmal differentiation, whereas expert pathological review reportedly favored BCC. Histopathological examination of a surgically excised left axillary lymph node in March 2026 subsequently demonstrated metastatic basaloid carcinoma with infiltration of the nodal capsule. A separate tissue fragment was also infiltrated by basaloid carcinoma. These findings confirmed nodal metastatic involvement but did not, in isolation, establish histological transformation or a distinct tumor subtype. Accurate histological classification is essential because treatment selection depends on the underlying diagnosis [1].

The initial use of vismodegib reflected the multidisciplinary determination that curative surgery and definitive radiotherapy were not feasible. The pivotal ERIVANCE study established the activity of vismodegib in locally advanced and metastatic BCC [2]. Earlier phase I findings and the subsequent larger STEVIE (SafeTy Events in VIsmodEgib) trial also supported Hedgehog pathway inhibition as a systemic treatment approach for advanced BCC [5,6]. However, disease progression during treatment remains a clinically important limitation. Genomic investigations have linked resistance to reactivation of Hedgehog signaling, particularly through alterations in smoothened and, less frequently, downstream pathway components [7]. These mechanisms provide a biological context for progression during vismodegib treatment, although no molecular testing was performed to establish the mechanism in this patient. Clinical progression was reported after approximately five months of treatment. Because an initial objective response assessment was unavailable, the course cannot be classified confidently as acquired resistance.

The most notable imaging finding during second-line treatment was the substantial reduction in tumor volume on the December 2025 MRI compared with the June 2025 examination following the initiation of cisplatin and 5-fluorouracil. Regression involved both the superficial disease and the deeper parotid and pharyngeal components. Historical evidence of activity with cisplatin-based chemotherapy in advanced cutaneous BCC and squamous cell carcinoma provides context for this observation [8]. However, this evidence derives from a small mixed-histology series using predominantly cisplatin and doxorubicin and should not be interpreted as direct validation of cisplatin and 5-fluorouracil in BCC.

The MRI findings documented radiographic improvement during the second-line treatment period. Nevertheless, there was an absence of standardized lesion measurements, complete chemotherapy records, and detailed assessment of the effects of other treatment exposures, which limits causal attribution and prevents assignment of a formal Response Evaluation Criteria in Solid Tumors (RECIST) response category. This observation cannot establish the general efficacy of the regimen in advanced BCC. Current European guidance prioritizes Hedgehog pathway inhibition followed by anti-programmed cell death protein 1 therapy in appropriate patients [1]. The clinical rationale for selecting cytotoxic chemotherapy in this patient was also not fully documented, which limits evaluation of this part of the treatment sequence.

The March 2026 PET/CT demonstrated a more complex response pattern. Metabolic activity decreased in the left tonsillar finding and several nodal regions, while the left facial and cervical soft-tissue tumor became substantially smaller despite an increase in SUVmax. Newly apparent metabolically active right axillary lymph nodes were also identified. These findings support the description of a heterogeneous response. The newly apparent right axillary lymph nodes were not sampled and therefore remained histologically unconfirmed. In contrast, subsequent surgical biopsy of a persistent left axillary lymph node provided histological confirmation of metastatic basaloid carcinoma. Changes in SUVmax alone were insufficient to assign a formal overall metabolic response category. Furthermore, comparison with the February 2025 examination encompassed treatment with vismodegib, palliative radiotherapy, and chemotherapy, preventing isolation of the contribution of any individual treatment. The left tonsillar finding and the earlier indeterminate second-rib focus should likewise remain distinguished from histologically confirmed metastatic disease. This complexity reinforces the importance of longitudinal assessment integrating clinical examination, symptoms, and imaging, consistent with practical recommendations for monitoring advanced BCC during Hedgehog pathway inhibitor treatment [9].

Cemiplimab has demonstrated antitumor activity in both locally advanced and metastatic BCC following Hedgehog pathway inhibitor therapy [3,4]. In this case, only one cycle was administered before the patient died, and no subsequent response assessment was available. The clinical course therefore provides no basis for concluding that cemiplimab was ineffective or that its administration contributed to the patient’s death. Interpretation also requires confirmation of the patient’s performance status and overall clinical condition at treatment initiation, which should not be inferred from the ECOG performance status of 3 documented during the subsequent palliative-care admission.

The later clinical course was characterized by ulcerated lesions, severe pain, poor oral intake, anemia, hypoalbuminemia, and functional decline. These findings emphasize the need to evaluate treatment benefit alongside symptom burden, nutritional status, and functional capacity and to integrate supportive and palliative care throughout disease management. Although treatment-related toxicity may have contributed to the overall clinical burden, the available records do not allow its contribution to be separated reliably from that of progressive disease or other causes.

Limiations

There are a number of limitations. The original histopathology and immunohistochemistry reports were not available. A formal RECIST response category was not available. The date of cemiplimab administration, her clinical condition immediately before treatment, the interval between treatment and death, and the documented cause of death could not be verified from the available records. While seven cycles of chemotherapy with cisplatin and 5-fluorouracil were administered, the exact treatment dates, doses, intervals, and any dose modifications could not be verified from the available documentation. Incomplete pathology records, the lack of comparative histological and immunohistochemical review, and gaps in treatment details and standardized response measurements limit assessment of histological changes and treatment-specific outcomes. Limited documentation of the final treatment and death also prevents firm conclusions about the causes of clinical deterioration.

However, the documented clinical course illustrates the diagnostic and therapeutic challenges of managing advanced BCC through successive lines of systemic therapy. Despite the limitations, the case illustrates the diagnostic uncertainty and therapeutic complexity of advanced BCC while emphasizing the importance of distinguishing radiographic improvement at individual disease sites from sustained overall disease control and clinical benefit.

## Conclusions

This case illustrates the diagnostic and therapeutic challenges associated with advanced BCC of the head and neck. Substantial radiographic regression was documented during second-line treatment with cisplatin and 5-fluorouracil after progression during vismodegib therapy. However, subsequent heterogeneous imaging findings, histologically confirmed lymph-node involvement by basaloid carcinoma, and clinical deterioration underscored the distinction between site-specific tumor regression and sustained overall clinical benefit. The differing pathological descriptions highlight the importance of expert pathological review and diagnostic clarification. Only one cycle of cemiplimab could be administered before the patient’s death, precluding assessment of its efficacy. Management of such cases requires multidisciplinary evaluation, longitudinal clinical and imaging assessment, and integration of symptom control and supportive care throughout the treatment course.
